# High Prevalence of Cardio-Metabolic Risk Factors in a Young Urban Sri-Lankan Population

**DOI:** 10.1371/journal.pone.0031309

**Published:** 2012-02-13

**Authors:** Mahen Wijesuriya, Martin Gulliford, Judith Charlton, Laksha Vasantharaja, Giancarlo Viberti, Luigi Gnudi, Janaka Karalliedde

**Affiliations:** 1 Diabetes Association of Sri Lanka, National Diabetes Centre, Colombo, Sri Lanka; 2 Cardiovascular Division, King's College London, London, United Kingdom; 3 Division of Health and Social Care Research, King's College London, London, United Kingdom; University of Perugia, Italy

## Abstract

**Background:**

South-Asian's are predisposed to early onset type 2 diabetes (T2DM). The prevalence of cardio-metabolic risk-factors in young Sri-Lankans is unknown.

**Methodology/Principal Findings:**

To determine by questionnaire and anthropometry the prevalence of first degree family history (FH) of T2DM, physical inactivity, raised waist circumference (WC) and raised body mass index (BMI) in a representative healthy urban population selected by cluster sampling. Those with ≥2 risk-factors were evaluated for metabolic syndrome (MS) and recruited for an intervention trial. Of 23,296 participants screened, 22,507 (53% Female) were eligible [8,497 aged 10–14 yrs, 4,763 aged 15–19 yrs and 9,247 aged 20–40 yrs]. 51% had none of the 4 risk-factors, 26% 1 risk-factor and 23% (5,163) ≥2 risk-factors of whom 4,532 were assessed for MS. Raised BMI was noted in 19.7% aged 10–14 yrs, 15.3% between 15–19 yrs, and between 20–40 yrs, 27.4% of males vs. 21.8% of females p<0.001. Prevalence of raised WC was greater in females for each age group: 42.7% vs. 32.1%; 28.1% vs. 16.1%; 34.5% vs. 25.7% (p<0.05 for all) as was physical inactivity: 39.9% vs. 14.5%; 51.7% vs. 20.0%; 62.7% vs. 41.3% which rose in both sexes with age (p<0.05 for all). FH of T2DM was present in 26.2%. In 4532 (50%<16 yrs) with ≥2 risk-factors, impaired fasting glycaemia/impaired glucose tolerance (pre-diabetes) prevalence was 16%. MS was more prevalent in males [10–16 yrs (13.0% vs. 8.8%), 16–40 yrs (29.5% vs. 20.0%) p<0.001 for both].

**Conclusions/Significance:**

There is a high prevalence of modifiable cardio-metabolic risk-factors in young urban Sri-Lankans with significant gender differences. A primary prevention intervention trial is ongoing in this cohort. Clinical Trial Registration Number SLCTR/2008/World Health Organization (WHO) international clinical trial registry platform.

## Introduction

Type 2 Diabetes Mellitus (T2DM) and associated cardiovascular complications pose a major health care burden worldwide. Recent data indicates that South Asia is one of the major sites of this epidemic of T2DM with a projected 72% increase in the number of subjects with T2DM in the next 20 years [1]. In parallel there is an epidemic of pre-diabetes [impaired glucose tolerance (IGT) and impaired fasting glycaemia (IFG)] with prevalence rates between 10–15% reported in South Asian adult populations [1]. T2DM and IGT are associated with a significantly increased risk of cardiovascular disease (CVD) with South Asian's particularly predisposed to early onset of T2DM and CVD, with almost a third of future T2DM cases predicted to be in those below 45 years [1]. Both CVD and T2DM may share a common pathogenesis and indeed retain many common risk factors/features [1], [2].

Sri-Lanka is a middle income country in Asia with a population of approximately 20 million. Nearly 40% of Sri Lankans are aged below 40 years and 25% are aged below 18 years [3]. There has been a rapid urbanisation in recent decades with an estimated 30% of the population now living in urban areas [3]. The most recent national study in subjects aged over 20 years indicated a population prevalence of dysglycaemia (defined as T2DM or IGT or IFG) of 20% which rose to 30% in urban areas [4]. Physical inactivity, raised body mass index (BMI) and central obesity along with urban living were strongly associated with the increased risk of dysglycaemia. In this study only 35% (n = 1530) of subjects were below 40 years of age of whom less 300 were urban dwelling and no subjects aged below 20 years were studied [4].

The DIABRISK-SL research programme is aimed to implement and evaluate a primary prevention intervention to reduce the incidence of T2DM and predictors of cardio-metabolic disease in young at-risk subjects. We employed a ‘high-risk’ strategy by selecting individuals who were at increased risk of T2DM or CVD. In view of the relatively young age of onset of these conditions in the Sri Lankan population, the project aimed to recruit males and females who were between 10–40 years old. The purpose of this paper is to report on the sampling strategy and screening procedures. Furthermore in view of the limited information available concerning cardio-metabolic risk in youthful South Asian populations, this report also aims to describe the frequency of cardio-metabolic risk factors in the screened population, as well as in the high risk sample.

## Methods

### Ethics statement

Ethical approval from the Sri-Lanka Medical Association Ethical Review Committee and permission from the Ministry of Education was obtained for this study which was conducted under the Good Clinical Practice principles and guidelines and according to the principles expressed in the Declaration of Helsinki for clinical research (Text S1). All study participants gave written informed consent.

DIABRISK-SL [Clinical Trial Registration Number SLCTR/2008/World Health Organization (WHO) international clinical trial registry platform] is an on-going open randomized controlled parallel group clinical trial evaluating the effect of intensive (3-monthly) vs. less-intensive (annual; control group) lifestyle modification advice on a primary composite cardio-metabolic end point (new onset T2DM and IGT, hypertension, albuminuria, CVD and renal events) in ‘at risk’ urban subjects aged 10–40 years. This manuscript presents the results from stage one (screening) and stage two (baseline evaluation of subjects ‘at high-risk’ selected for intervention) of the study.

### Subjects

Stage one involved screening of a population representative of the general population of Colombo District, the most populous urban district in Sri-Lanka, aged between 10 to 40 years to identify the prevalence of four risk-factors namely first degree family history (FH) of T2DM, physical inactivity, raised BMI and raised waist circumference. Stage 2 involved subjects with two or more risk- factors being invited to attend the National Diabetes Centre for baseline evaluation and characterization by clinical and laboratory testing prior to randomization into the two trial arms.

The sampling strategy was designed to provide a sample that was representative of the age and sex distribution of the general population aged between 10–40 years in the Colombo district [3]. A list of schools, workplaces, universities, civil and community organizations/societies within a 30 km radius of the National Diabetes Centre in Colombo Sri Lanka that was representative of the organizations and population aged between 10 to 40 years obtained from the most recent national census results for the district of Colombo was made. This list comprised of 65 organizations and each of these organizations were individually approached and eligible participants were invited to take part in the screening study. Community organizations included societies and communities which included subjects who were unemployed, school drop outs, and housewives within the age group specified. The age and sex distribution of the screened population was regularly compared with the distribution of the reference population from the census data to ensure that the screened population was representative of the distribution of the general urban population aged between 10 and 40 years in the Colombo district. More than 95% of subjects and institutions approached agreed to participate. An open invitation via leaflet and posters for any member (within the age group criteria of the study) of these institutions was made and all those who volunteered and fulfilled the inclusion criteria were accepted into the study.

Screening was conducted by a research team of between 6–12 personnel from the National Diabetes Centre who are all trained in healthcare research methodology and practice. These staff were supervised and supported by one medical doctor and one clinical study coordinator.

Stage 1 took place on site at the respective institutions/sites and stage 2 was done at National Diabetes Centre in Colombo. The screening survey took place between 1^st^ January 2008 and 30^th^ June 2009. Subjects with a known history of T2DM, CVD, hypertension or diabetes and pregnant women were excluded from the initial sample. Subjects on lipid lowering drugs or anti-diabetic or anti-hypertensive drugs were also excluded. A screening questionnaire (Text S2) was used to determine first degree FH of T2DM and the physical activity of the individual participants. Further information regarding socio-demographic factors was also collected.

### Procedures

The screening study aimed to identify four risk-factors: physical inactivity (<30 minutes continuous exercise for <3 days/week), raised WC (central obesity) defined as WC in subjects between 10–17 yrs ≥91^th^ percentile, 18–40 yrs: females ≥80 cm and males ≥90 cm, first degree FH of T2DM and raised BMI defined in subjects aged 10–18 yrs of a BMI value greater than internationally standardized age and sex specific percentile cutoffs and between 18–40 yrs as BMI ≥23 kg/m^2^ [5], [6], [7]. These four factors were chosen on the basis of a pilot study that identified these risk-factors as being highly prevalent in 1239 subjects aged below 40 years with a new diagnosis of T2DM who attended the National Diabetes Centre in Colombo between 2005–2007 and are supported by recent data from comparable South Asian and Sri Lankan populations [1], [4], [7], [8].

Following the screening questionnaire anthropometric measurements of WC, height and weight were performed in all subjects. Height was measured using a portable stadiometer (Seca, Rumily, France) calibrated weekly and taken to the closest 0.1 cm. Weight was measured with an electronic weighing scale (Seca, Rumily, France), calibrated weekly, without footwear in light clothing to the nearest 100 g. BMI was calculated as weight in kilograms divided by height squared in meters. WC taken using a graduated measuring tape (Seca, Rumily, France), calibrated weekly, with a locking device to the nearest 0.1 cm. WC was taken at the mid- point between the iliac crest and the last rib in expiration. WHO approved growth charts for males and female children were used [9].

In stage 2 subjects with two or more risk factors attended the National Diabetes Centre where biochemical testing and a standard 2 hour 75 g oral glucose tolerance test (OGTT) was performed in adults and glucose solution 1.75 g/kg body weight, to a maximal dose of 75 g was given to those below 16 years following a 12 hour fast as per WHO guidelines [10]. Plasma Glucose, total cholesterol, high density lipoprotein (HDL), low density lipoprotein (LDL),triglycerides and serum creatinine were measured by enzymatic colorimetry (Vitros MS 250, Ortho Clinical Diagnostics, Johnson and Johnson, Rochester USA). Fasting plasma insulin was measured using radioimmunoassay (Immulite, Siemens, Surrey, UK), which has a sensitivity of 4 µU/ml (<24 pmol/l) and intra and interassay coefficients of variation <8%. Insulin resistance was calculated using the homeostasis model assessment of insulin resistance (HOMA-IR) [11]. Urine albumin to creatinine ratio was measured by immunoturbidimetry (Vitros 5,1 FS Chemistry System, Ortho-Clinical Diagnostics, Johnson and Johnson, Hampshire, United Kingdom) from an early morning urine sample. Blood pressure was measured by automatic oscillometry from the non-dominant arm after 5 minutes rest with the mean of two measurements recorded (Riester Ri-Champion Riester, Jungingen, Germany).

New cases of T2DM were diagnosed according to the WHO and American Diabetes Association criteria [10], [12]. IGT was defined as fasting glucose <126 mg/dl (7.0 mmol/l) and 2-h glucose 140–200 mg/dL (7.8–11.0 mmol/l) and IFG as fasting plasma glucose 100–125.9 mg/dL (5.6–7 mmol/l) [12]. New cases of hypertension were defined as mean brachial blood pressure ≥140/90 mmHg in adults and those below 18 years hypertension was defined as blood pressure that is, on repeated measurement, at the 95th percentile or greater adjusted for age, height, and sex as per Joint National Committee guidelines on Prevention, Detection, Evaluation, and the Treatment of High Blood Pressure (JNC-7) [13].

Metabolic syndrome, was defined by International Diabetes Federation (IDF) criteria for children, adolescents and adults [6], [14]. These are for subjects ≥16 years the presence of central obesity plus any two of the following; raised triglycerides levels ≥150 mg/dl (1.7 mmol/l), reduced HDL-cholesterol <40 mg/dL (1.03 mmol/l) in males and <50 mg/dL (1.29 mmol/l) in females, raised blood pressure [systolic blood pressure (SBP) ≥130 mmHg or diastolic blood pressure (DBP) ≥85 mmHg] and raised fasting glucose ≥100 mg/dL (5.6 mmol/l) is required [13]. The IDF criteria for those between 10 to 16 years are a central obesity plus the presence of any two or more of following; raised triglycerides, reduced HDL-cholesterol, raised SBP or DBP mmHg and raised fasting blood glucose [6].

The team involved in data collection followed good clinical research practice guidelines. All data entry was double checked by two researchers and entered into a dedicated computer soft ware data base system which was maintained and scrutinized by an in house IT consultant. The research team have monthly internal reviews of the data base and data entry processes/procedures to ensure that good practice is followed for data entry/data management. These process and procedures has been independently reviewed and approved by the independent advisory and data monitoring committee for the study and reviewers from the IDF Bridges grant committee.

### Statistical Analysis

Data values were coded to missing if they were more than four standard deviations from the sample mean. The number of such values was noted in 30 subjects. Descriptive statistics were used for the analysis of demographic and clinical features of the cohort. Analyses were performed using STATA software version 11 (Stata Corp, College Station, TX, USA). The cluster sampling design gave rise to substantial intraclass correlation coefficients. The ‘svy’ commands in Stata were utilized to obtain standard errors through Taylor-linearized variance estimation with organization as the primary sampling unit. Descriptive statistics were used for the analysis of demographic and clinical features of the cohort. Data are given as mean (95% confidence intervals) unless otherwise stated. Group comparisons were made using median test or chi squared test as relevant. All *P* values are presented with adjustment for multiple comparisons. Analyses were performed using STATA software version 10 (Stata Corp, College Station, TX, USA).

## Results

The sample was drawn from 65 organizations which included 38 workplaces, 15 schools, five universities and 7 community organizations. There were 23,296 subjects screened for the study. There were 393 subjects excluded because they were aged less than 10 years or greater than 40 years and 396 excluded due to being on treatment for and/or having a known diagnosis of T2DM, CVD, hypertension or raised cholesterol levels, kidney disease and pregnancy.

After excluding these ineligible subjects there were 22,507 participants (47% males, 53% female) evaluated in stage one of the study. Of the 22,507 eligible subjects 8,497 (males 44.8%) were aged 10 to 14 years, 4,763 (males 41.4%) aged 15 to 19 years and 9,247 (males 51.3%) aged 20 to 40 years. The clinical and anthropometric characteristics of the 22,507 subjects are presented in Table 1. The overall prevalence of a raised BMI between 10–14 and 15–19 yrs was 19.7% and 15.3% respectively with no significant differences between males and females. Between 20–40 years 27.4% of males and 21.8% of females were overweight (p<0.001 males vs. females). In contrast there was no significant gender differences in the prevalence of obesity in subjects aged between 20–40 years and below 14 years. Between 15–19 years the prevalence of obesity was significantly higher in males although the overall number of subjects were smaller than in the other age ranges (Table 1). Prevalence of central obesity was significantly greater in females for each age group (Table 1). Similarly physical inactivity was significantly greater in females compared to males for each age group (Table 1).

**Table 1:** Demographic and anthropometric characteristics of 22,507 subjects representative of the urban population of Colombo District aged between 10–40 years.

Characteristic	Age 10–14 (n = 8,497)		Age 15–19 (n = 4,763)		Age 20–40 (n = 9,247)	
Gender	Males (n = 3,802)	Females (n = 4,695)	Males (n = 1,974)	Females (n = 2,789)	Males (n = 4,741)	Females (n = 4,506)
Height (cm)	150.6 (149.0 to 152.1)	150.2 (148.5 to 152.0)	168.6 (167.2 to 168.5)	156.8 (156.3 to 157.3)	167.9 (167.2 to 168.5)	155.2 (154.6 to 155.8)
Weight (kg)	42.0 (40.2–43.7)	43.0 (41.0 to 45.0)	57.8 (55.9 to 59.8)	50.4 (49.5 to 51.2)	64.4 (62.7 to 66.2)	53.1 (51.1 to 55.1)
BMI (kg/m^2^)	18.2 (17.7 to 18.7)	18.8 (18.3 to 19.3)	20.3 (19.8 to 20.7)	20.4 (20.1 to 20.8)	22.8 (22.3 to 23.4)	22.0 (21.3 to 22.7)
Waist (cm)	67.9 (66.3 to 69.4)	66.9 65.2 to 68.5)	75.5 (73.7 to 77.3)	70.6 (69.3 to 72.0)	83.0 (81.4 to 84.5)	75.4 (72.8 to 78.1)
FH of T2DM	21.0 (18.8 to 23.2)	23.8 (20.9 to 22.7)^b^	26.1 (22.4 to 29.8)	30.8 (29.3 to 32.3)^b^	27.0 (22.6 to 31.3)	29.9 (25.2 to 34.5)^b^
Physical inactivity	14.5 (12.6 to 16.3)^c^	39.9 (29.5 to 50.1)^b^ ^c^	20.0 (16.2 to 23.7)^c^	51.7 (46.4 to 56.9)^b^ ^c^	41.3 (34.8 to 47.6)^c^	62.7 (51.7 to 73.7)^b^ ^c^
Overweight	19.0 (15.2 to 22.8)	20.2 (17.2 to 23.2)	15.5 (12.2 to 18.7)	15.2 (12.3 to 18.2)	27.4 (22.2 to 32.6)^a^	21.8 (16.3 to 27.4)
Obese	4.7 (3.2 to 6.1)	4.3 (3.1 to 5.6)	4.0 (2.8 to 5.1)	2.7 (1.9 to 3.4)	3.8 (2.6 to 5.0)	4.2 (2.0 to 6.3)
Central obesity	32.1 (27.0 to 37.3)	42.7 (37.0 to 48.4)^b^	16.1 (9.7 to 22.4)^b^	28.1 (22.0 to 34.2)^b^	25.7 (20.2 to 31.1)^b^	34.5 (25.2 to 43.8)^b^

a males vs.females p<0.001.

b females vs.males p<0.05.

c Age 10–14 years vs. 15–19 yrs vs 20–40 yrs p<0.05 for all.

Data are reported as means or proportions (%) with 95% confidence intervals corrected for cluster sampling.

Abbreviations: BMI, body mass index; FH, first degree family history; T2DM, type 2 diabetes mellitus.

Furthermore physical inactivity significantly increased with age for both males and females (p<0.01). A first degree FH of T2DM was documented in 26.5% of all subjects studied. Of interest a FH of T2DM was significantly more prevalent in females compared to males across all age groups (p<0.01).

Of the eligible subjects 11,456 (51%) had none of the four risk factors, 5,888 (26%) one risk factor and 5,163 (23%) two or more risk factors. Two or more risk factors were noted in 24.0% of subjects aged 10–14 yrs, 16.9% between 15–19 yrs and 25.0% in those aged 20–40 yrs.

Of the 5,163 subjects with two or more risk factors 4532 subjects (50% below 16 years) were further evaluated in stage 2 and entered the prospective clinical trial. Those subjects eligible for participation in the trial following screening but who did not wish to take part or failed attend for further evaluation (n = 631) did not differ from those entered into the clinical trial in any baseline characteristics (data not shown).

The clinical and biochemical baseline characteristics of the 4,532 selected subjects according to age groups and gender are shown in Table 2. In the whole cohort central obesity was detected in 70.6% of subjects and a raised BMI in 51.1%.

**Table 2:** Baseline clinical and biochemical characteristics of 4532 subjects aged between 10–40 years with ≥2 cardio-metabolic risk factors selected for participation in the randomized controlled trial.

Characteristic	Age 10–14(n = 1,382)		Age 15–19(n = 822)		Age 20–40(n = 2,328)	
Gender	Males (n = 596)	Females (n = 786)	Males (n = 260)	Females (n = 562)	Males (n = 1,212)	Females (n = 1,116)
BMI (kg/m^2^)	22.8 (22.2 to 23.4)	21.8 (21.0 to 22.6)	25.1 (24.5 to 25.7)	23.3 (23.0 to 23.7)	25.7 (25.4 to 26.0)	24.8 (24.2 to 25.3)
Waist circumference (cm)	82.5 (80.6 to 84.4)	76.0 (73.6 to 78.4)	89.5 (87.7 to 91.3)	79.7 (78.6 to 80.8)	91.6 (90.6 to 92.7)	84.8 (83.2 to 86.3)
SBP (mmHg)	108 (106 to 110)	108 (107 to 109)	119.1 (118 to 120)	111 (109 to 113)	123 (122 to 124)	113 (112 to 114)
DBP (mmHg)	66 (65 to 67)	67 (66 to 68)	71 (70 to 73)	69 (68 to 71)	77 (77 to 78)	72 (71 to 73)
FPG (mg/dL))	90 (89 to 90)	88 (87 to 89)	90 (89 to 90)	88 (87 to 89)	94 (92 to 95)	90 (88 to 93)
2 hr OGTT PG (mg/dL)	103 (102 to 105)	105 (104 to 108)	102 (99 to 105)	106 (103 to 108)	113 (109 to 117)	115 (109 to 121)
Triglycerides (mg/dL)	107 (104 to 110)	97 (90 to 105)	106 (97 to 115)	88 (83 to 92)	147 (141 to 152)	101 (98 to 105)
Total cholesterol (mg/dL)	181 (178 to 183)	179 (177 to 181)	174 (172 to 177)	182 (179 to 185)	210 (208 to 213)	196 (195 to 198)
LDL cholesterol (mg/dL)	114 (111 to 116)	113 (111 to 114)	111 (110 to 113)	116 (113 to 119)	140 (138 to 143)	130 (128 to 131)
HDL cholesterol (mg/dL)	45 (44 to 46)	47 (45 to 48)	42 (40 to 43)	48 (47 to 49)	40 (39 to 41)	46 (45 to 47)
Fasting insulin (mIU/L)	14.2 (12.4 to 16.0)	14.0 (12.6 to 15.5)	14.4 (13.1 to 15.8)	12.9 (12.2 to 13.5)	11.1 (10.3 to 11.8)	10.1 (9.8 to 10.4)
HOMA-IR %	3.1 (2.8 to 3.5)	3.1 (2.8 to 3.4)	3.2 (2.9 to 3.5)	2.8 (2.7 to 3.0)	2.6 (2.4 to 2.8)	2.3 (2.2 to 2.4)
Serum creatinine (mg/dL)	0.59 (0.57 to 0.61)	0.56 (0.55 to 0.57)	0.80(0.77 to 0.82)	0.64 (0.63 to 0.65)	0.96 (0.94 to 0.97)	0.69 (0.68 to 0.70)
Urine ACR (mg/g)	6.0 (5.1 to 6.9)	10.8(6.7 to 15.0)	8.4 (3.9 to 12.8)	8.9 (5.9 to 11.9)	6.0 (4.0 to 8.1)	8.7 (5.7 to 11.7)

Data are reported as means with 95% confidence intervals corrected for cluster sampling.

Abbreviations: BMI, body mass index; SBP, Systolic blood pressure; DBP Diastolic blood pressure; FPG, Fasting plasma glucose; PG, plasma glucose; LDL, low density lipoprotein; HDL, high density lipoprotein; HOMA-IR, Homeostasis model assessment for insulin resistance; ACR, albumin to creatinine ratio.

In those below 14 years, 29 (4.7%) males and 31 (3.9%) females were diagnosed with IFG. In those between 15 to 19 years, 24 males (9.2%) and 17 females (3.0%) had IFG. IFG was diagnosed in 190 (15.1%) males and 90 females (7.9%) between 20–40 years. Prevalence of IGT was identical in males and females below 14 years (3.9% for both 31 females and 24 males), and was similar between males and females aged between 14–19 years [25 (4.4%) females and 11 (4.2%) males]. Between 20–40 years, 148 males (11.7%) and 123 (10.8%) females had IGT.

Overall prevalence of IFG and IGT was 16%. T2DM was newly diagnosed in 106 subjects (59 males and 47 females) with 3 cases (all females) in subjects below 15 years and 6 cases (5 females, 1 male) in subjects between 15–19 years of age and the remainder in those between 20–40 years (58 males, 39 females). Fasting insulin levels, HOMA-IR were not significantly different between males and females in the reported age ranges (Table 2). Urine ACR results confirmed well known gender differences and ACR did not increase significantly with age.

As the age specific IDF criteria to diagnose metabolic syndrome use 16 years as a cut off, the 4532 selected subjects were split in to two age groups (above and below 16 years) for further analyses.

In subjects below 16 years prevalence of central obesity was significantly greater in males vs. females (88.5% vs. 79.2% p<0.001). This trend was reversed in those over 16 years with a greater prevalence of central obesity in females (67.4% vs. 59.9% p<0.001). Elevated triglycerides were noted in 12.2% of subjects below 16 years and 24.5% above 16 years. Males had a greater prevalence of elevated triglycerides as compared to females in subjects below 16 years (15.0% vs. 10.0% p = 0.002) and above 16 years (36.6% vs. 12.8% p<0.001). Low HDL levels were detected in 28.3% of those below 16 years and in 58.9% of subjects over 16 years of age. Below 16 years males had a significantly greater prevalence of low HDL levels (32.8% vs. 25.2% p = 0.001) and over 16 years this trend was reversed with a greater prevalence in females (50.8% of males vs. 66.7% of females p<0.001). Raised blood pressure was detected in 6.7% of those below 16 years and in 23.1% of subjects above 16 years of age. There were no significant gender differences in the prevalence of hypertension in those below 16 years (7.2% of males vs. 6.2% of females). Above 16 years males had a significantly greater prevalence of hypertension (33.2% vs. 13.4% p<0.001).

In subjects below 16 years of age the prevalence of raised fasting plasma glucose was similar between males and females (5.9% of males vs. 4.2% of females), however over 16 years males had a greater prevalence (17.2% of males vs. 8.5% of females p<0.001). Metabolic syndrome was diagnosed in 13.0% of males and 8.8% of females below 16 years (p = 0.005) and in 29.5% of males and 20.0% of females over 16 years (p<0.001).

## Discussion

This is the first large population based study to determine the prevalence of T2DM, pre-diabetes (IGT and IFG), and cardio-metabolic risk factors in young urban Sri-Lankans. Our results show a high prevalence of modifiable risk factors for cardio-metabolic disease in subjects aged between 10–40 years. Importantly these risk factors are present early with significant gender and age differences. We also aimed to develop a method for identifying a sample at increased risk of diabetes and cardiovascular disease for a randomized controlled trial of a new intervention strategy. The results show that a simple screening strategy based on self-reported physical inactivity, family history of diabetes in first degree relatives, as well as obesity and increased waist circumference provides an efficient method for identifying a sample with a very high frequency of features characteristic of the metabolic syndrome. Recently the entity of the metabolic syndrome per se and its value as syndrome has been questioned [15]. What is relevant is that the metabolic syndrome does identify subjects at higher cardio-metabolic risk and that the individual risk factors for CVD and T2DM cluster and are inter-related [14]–[16]. Metabolic syndrome is a practical tool in focusing public health attention and in its purest form, as we have reported with the exclusion of those with known T2DM or CVD and with a detailed description of the individual and related risk components, it is a pre-morbid condition that may be useful in developing country specific public health intervention strategies [14], [15].

In this study we report the early determinants of cardio-metabolic risk in a young at risk population and this work will helps us further understand underlying key mechanisms such as central obesity and insulin resistance, which underlie the clustering of cardio-metabolic risk factors [14].

Our results from the screening of a sample representative of the general population of Colombo district, indicate that in children aged between 10–14 years two or more risk factors for cardio-metabolic disease were noted in 24% which is nearly equivalent to the prevalence in those >20 years. This has not been reported previously in Sri Lanka or South Asia and is of great public health concern. The exact mechanisms/explanations for this high prevalence remain unclear however we could speculate that recent trends in less school time exercise/sports and increased exposure of children to unhealthy diets may underlie these results. In children aged between 10 to 14 years the prevalence of central obesity was 38% and nearly 20% were overweight with a significantly greater prevalence of central obesity in females. In subjects between 15–19 years the prevalence of central obesity was 23.1% with a greater female preponderance. Our data are similar to that reported in 2640 subjects aged between 12–19 years from Chennai, South India where prevalence of overweight subjects was 15% and central obesity defined by a lower waist circumference cut-off (>75th percentile) was noted 23.7% subjects [16]. The authors of this study however did not report significant gender differences in their results. A recent study from the city of Karachi in Pakistan reported an overall prevalence of overweight and obesity of 7% in boys and 11% in girls aged 13–14 years which the authors reported is a two fold rise over 10 years [17]. This dramatic rise was associated with a decline in physical activity and this data as well our results mirrors global trends of rising childhood obesity. The prevalence of obesity we report in those below 14 years is broadly similar to that reported from much smaller studies in Sri-Lanka, however in contrast to previous reports work we did not observe a significantly higher prevalence in males [18]. Importantly being overweight in childhood is an antecedent of adult obesity and is associated with heightened risk of overt cardio-metabolic disease. Therefore, it is vital to estimate the prevalence of overweight/obese children as it is a step towards identifying high risk groups to evolve preventive strategies.

Recent data from adults subjects from Pakistan and Sri Lanka indicate that the factors independently and significantly associated with being overweight and obesity include older age, being female, urban residence, being literate, and having a high economic status [19], [20].

The percentage prevalence of overweight adults (defined as BMI≥23 kg/m^2^) and obese (defined as ≥BMI 27.5 kg/m^2^) urban Sri-Lankans in a recent national survey was 32.7% and 18.5% respectively with females noted to have a greater prevalence in both categories compared to males [19]. Our data from a younger population indicates similar overall prevalence of overweight subjects but in contrast to the above results our results in those aged between 20–40 years show a greater prevalence of being overweight and obese in males. This may be explained by important differences in the subjects included in the two studies. Subjects assessed in the national survey, were significantly older (mean age 46.3 years) than those in our study and the number of urban subjects studied was smaller (n = 1530). Our data confirms previous results which show a higher prevalence of central obesity in females than males aged between 20–40 years, although the overall prevalence in urban males we report of 25.7% is higher than the 15% prevalence described nationally [18].

Overall 26.2% of subjects had a first degree family history of T2DM which was significantly more prevalent in females across all age groups. The prevalence of parental history of T2DM has not previously been studied in detail in young urban Sri-Lankans and prospective studies are required to explain the possible significance of the above results. A smaller study in South Asians subjects (n = 321) has previously demonstrated increased risk of glucose intolerance, being overweight, low levels of HDL and high blood pressure in Asian Indian adolescents with a family history of T2DM [21] but the relative contributions, if any, of maternal or paternal family history were not described.

In our ‘high-risk’ cohort of subjects with 2 or more risk factors the commonest cardio-metabolic abnormality detected was low HDL cholesterol, followed by elevated triglycerides. Similar findings have been reported from South Asian adults and children [16] however as the value used to define raised triglycerides in those below 16 years in our study was higher than the values of 105 mg/dl (1.2 mmol/l) used in recent studies from South Asia [9] and western countries [21], this is likely to be an underestimation. Metabolic syndrome was detected in 10.4% of subjects <16 years and in 24.7% of those ≥16 years of age. Data on prevalence of metabolic syndrome and its individual components have not previously been reported in large numbers of young urban or general Sri Lankan populations. Our prevalence data for metabolic syndrome in children and young adults are higher than reports from recent studies in other countries [9], [22], [23] and this difference is likely to be due to our selection of subjects at ‘high-risk’ in the context of screening and selection strategy to detect and recruit several thousand ‘high-risk’ subjects for a primary prevention trial. Interestingly the metabolic syndrome prevalence we report of 11% is significantly lower than that of 21% reported in a smaller number of children attending a specialist obesity clinic in Colombo, Sri Lanka [24].

A limitation of our study was that we could not obtain full details and data on a family history of obesity in the subjects screened. However this study was not primarily designed to look for these associations. Another limitation was that we could not formally assess pubertal stages in children when they were screened. However in the context of a population based large screening study and local cultural attitudes this was not a feasible.

The strengths of our study is that, it is the largest urban cohort below the age of 40 years in South Asia evaluated for cardio-metabolic risk with more than 50% of subjects being below 16 years a group hitherto not studied in detail or in such numbers. We provide novel information on the prevalence of key risk-factors for cardio-metabolic disease in young subjects from the Colombo District the most populous and urban district in Sri Lanka.

Our results which show a 16% prevalence of pre-diabetes states (IFG/IGT) in an at risk young population raises awareness of the growing burden of risk of T2DM and obesity in developing countries that is of major public health and economic importance and which requires urgent remedial actions [25], [26].

A prospective study that evaluates the effect of a non-pharmacological life style modification primary intervention programme on a primary composite cardio-metabolic end point and a range of cardio-metabolic secondary end-points in this young ‘high-risk’ population of urban subjects is ongoing. This study will also provide further information on the natural history of cardio-metabolic risk in this unique at risk cohort.

## Supporting Information

Text S1 Letter of ethical approval for the study.
(PDF)

Text S2 Screening questionnaire used to determine first degree FH of T2DM, physical activity of the individual participants and to enter height, weight and waist circumference measurements.
(PDF)
