# Supplementary material for: High Prevalence of Cardio-Metabolic Risk Factors in a Young Urban Sri-Lankan Population
Source: PLoS One. 2012 Feb 13;7(2):e31309. doi: 10.1371/journal.pone.0031309 (PMC3278419; doi:10.1371/journal.pone.0031309)
Supplement: Text S1 — Letter of ethical approval for the study. (PDF) [file pone.0031309.s001.pdf]

Approval from the Sri Lanka Medical Association-Ethics Review Committee

SLMA SRI LANKA MEDICAL ASSOCIATION
6, WIJERAMA MAWATHA, COLOMBO 7, SRI LANKA T'phone: 2693324 Fax: 2698802

10th August 2007

Dr Mahen Wijesuriya
National Diabetes Centre
50, Sarana Mawatha
Rajagiriya

Dear Dr Wijesuriya

ERC/07- 010: Evaluation of risk factors in the development of type 2 diabetes and cardiovascular disease in a young urban population in Sri Lanka

Thank you for submitting the revised informed consent forms and the letters from the Ministries of Education and Higher Education

Approval is hereby given for you to conduct the above research project

Yours sincerely

Prof Anoja Fernando
Chairperson
Ethical Review Committee

RECEIVED
1 5 AUG 2007
